# Public awareness and knowledge of the National Health Insurance in South Africa

**DOI:** 10.11604/pamj.2015.22.19.6131

**Published:** 2015-09-09

**Authors:** Geoffrey Setswe, Samson Muyanga, Jacqueline Witthuhn, Peter Nyasulu

**Affiliations:** 1 Human Sciences Research Council, 134 Pretorius Street, Pretoria, South Africa; 2 Department of Health Studies, University of South Africa, P.O. 392, UNISA, 0003, Republic of South Africa; 3 Department of Public Health, School of Health Sciences, Monash, South Africa; 4 School of Public Health, Faculty of Health Sciences, University of the Witwatersrand, Johannesburg, South Africa

**Keywords:** Awareness, knowledge, National health insurance, South Africa

## Abstract

**Introduction:**

Individuals residing in Limpopo, KwaZulu-Natal and the Eastern Cape provinces who had access to public health services were surveyed to determine public knowledge and awareness of the new National Health Insurance (NHI).

**Methods:**

A descriptive cross-sectional study was conducted and a total of 748 adult respondents were sampled using a two-stage systematic sampling design. Data were collected using mobile phone assisted personal interviews.

**Results:**

The study found that 80.3% of the respondents were aware of the NHI and slightly less than half (49.8%) of the respondents did not have knowledge of how the NHI works and 71.8% lacked awareness about the origin of the development of the NHI concept in South Africa. The knowledge of what the NHI would pay for was poor and 48.1% knew that the NHI Fund would pay for medical expenses if a person got sick and 45.7% knew that with health insurance, basic health requirement is ensured and that if one becomes ill, medical treatment would be paid for by the NHI Fund, 50.9% of respondents did not understand how the NHI Fund will pay for health care received, only 44.8% understood how the NHI will pay for health care services received.

**Conclusion:**

The public education campaigns to increase knowledge and understanding of the NHI scheme might have been inadequate hence might not have penetrated many communities. It is recommended that a comprehensive community consultation plan be established to increase awareness and knowledge of the NHI among community members targeting clinics, schools, pension pay points and other community sites.

## Introduction

South Africa has commenced a major transformation process of its health policy which will see the implementation of the National Health Insurance (NHI) Scheme to guarantee a universal access to health care for all its citizens. The NHI is expected to be rolled out over a 14 year period beginning from 2012 with 10 pilot sites in the country's nine provinces. The principles for establishing the NHI were described in the Green Paper, mainly centred on improving access to quality health services and to providing financial risk protection against health-related catastrophic expenditures [1]. The Green Paper on the NHI was formally published in August 2011 as a consultation document highlighting key elements of the scheme. Members of the public and key stakeholders were allowed to comment and contribute to how they want the scheme to be implemented [2, 3]. After the first 18 months since the launch of the NHI Green Paper, a report indicated that over a 100 submissions of comments and suggestions were received by the Department of Health, from medical scheme administrators, labour, pharmaceutical industry, professional associations for various occupations, statutory bodies, government departments, academia, civil society and the Parliament [1]. Recent studies have revealed that, due to inadequate knowledge about the NHI concept, low income households are initially unwilling to subscribe to such health insurance schemes because they do not believe in paying for services they may not use [4]. Poor government's track record of mismanagement and poor service delivery were main reasons why the general public in Kenya was sceptical about the proposed National Social Health Insurance Scheme (NSHIS). There were also fears that previous corrupt officials linked to the National Social Security Fund (NSSF) and National Health Insurance Fund (NHIF) would manipulate the new scheme for their gain [5]. The degree to which knowledge influences utilisation, acceptability and smooth implementation of health care interventions has been investigated previously [6–8]. Implementing a new system is no exception to the above. As in the introduction of any new system or change, the fear of unknown and protection of interests create anxiety among stakeholders and this may lead to poor uptake and acceptability of such a new system [9]. In order to determine public awareness and knowledge around the NHI scheme among South Africans, measurements of public awareness and knowledge from the two years NHI experience since the launch of the Green paper and the one year experience of ten pilot sites were used as a benchmark. In this paper, we report the findings of a post-test survey.

## Methods

**Study design:** a cross-sectional survey was conducted in June 2013 on adult residents using health services in three provinces of South Africa. This approach allowed measuring participant's awareness and knowledge of the new unfolding NHI scheme, thus providing a “snapshot” of the level of awareness and knowledge of the NHI at a significant point in time, after the release of the Green Paper and launch of pilot sites. This investigation was derived from a larger impact study of the Monash-Oxfam NHI project involving adult residents from the three provinces using a pre-test and post-test quasi-experimental community-based design. Participants provided written informed consent prior to the interview. Ethical approval was granted by Monash University Research and Ethics Committee.

**Study sites and population:** the study was conducted in Bela-bela (Limpopo province), Edendale ( KwaZulu-Natal province) and Nelson Mandela Metropolitan area (Eastern Cape Province). The Edendale Hospital in the Umgungundlovu district in KwaZulu-Natal is a NHI pilot site while the other two sites are not. Bela-bela and Edendale are predominantly rural towns while Nelson Mandela Metro is a mixture of urban, peri-urban and rural area near Port Elizabeth. These areas serve a population of approximately 55,000, over 600,000 and about 1,000,000 people, respectively [10].

**Sampling and sample size:** the three residential areas were each systematically subdivided into 3 distinct clusters. Respondents were selected by using a 3 stage cluster sampling design. A total of 100 respondents were randomly selected from each of the 3 clusters, amounting to a sample population of 300 in each of the three areas. Subsequently, a total of 900 individuals were selected using a two-stage systematic sampling design from the three provinces.

**Instrument description and data collection:** a questionnaire was designed and distributed by the National Department of Health. It was based on a question-and-answer information brochure derived from the NHI document. The questionnaire tested awareness, knowledge levels and understanding of the NHI and the same questionnaire was used in the pre and post-test surveys. Data collection was achieved by using mobile phone assisted personal interviews (MPAPI). Mobile-phone-assisted questionnaires were completed with selected respondents and submitted over the mobile network covering South Africa to an online data store. Only three of the submitted surveys were unaccounted. Respondents were identified through the capturing of a unique numeric identifier.

**Statistical analysis:** data analysis from the survey was facilitated by using SPSS (version 21.0). Outliers and other inconsistent variables were identified through completion of frequencies and cross tabulations. Chi-square test was used to test for differences in groups and t-test for differences in means. P≤.05 was considered statistically significant. Pre- and post-test questionnaires were matched for each respondent to assess the change in knowledge and understanding of the NHI and the state on implementation of the NHI. Data analysis, grading and calculation of percentages for correct answers for each correspondent were conducted. The percentage level of awareness, knowledge and understanding of NHI was analysed for each set of questions and answers provided.

## Results

**Demographic analysis**: Table 1 shows the characteristics of respondents that participated in the survey. From 900 recruited respondents, 748 respondents were used for the survey giving a response rate of 83.1%. Respondents comprised 212 (28.3%) from Limpopo, 220 (29.4%) from KwaZulu-Natal and 316 (42.2%) from the Eastern Cape. There were more female (62.1%) than the male (37.9%) respondents and 84.6% of the respondents were in the age range of 20-59 years while 7.5% were less than 20 years of age and 7.8% were 60 years and older. The majority of respondents, namely 73.4% were single and 21% were married. The study was conducted in townships where an overwhelming majority of respondents were Africans (98.1%) and only 1.9% were a combination of Whites, Coloureds or Indians. About three quarters (75.6%) were unemployed while only one-quarter (24.4%) of respondents were employed. While 4% of respondents said they had no education, 84.1% had between a primary education and a grade 12 education and 11.9% had a post-secondary school education Table 1.

**Table 1:** Demographic characteristics of the study participants

Variables	Frequency	Percent
**Participants**		
Limpopo	212	28.3%
KwaZulu-Natal	220	29.4%
Eastern Cape	316	42.2%
**Gender**		
Male	296	37.9%
Female	486	62.1%
**Age group**		
Less than 20 years	59	7.5%
20-29 years	292	37.3%
30-39 years	163	20.8%
40-49 years	124	15.9%
50-59 years	83	10.6%
60 years and older	61	7.8%
**Marital status**		
Single	574	73.4%
Married	164	21%
Divorced	16	2%
Widowed	28	3.6%
**Racial group**		
African	767	98.1%
White, Coloured and Indian	16	1.9%
**Employment status**		
Employed	191	24.4%
Unemployed	591	75.6%
**Educational level**		
No education	31	4%
Primary education	116	14.8%
Grade 10	239	30.6%
Grade 12	303	38.7%
Tertiary	64	8.2%
Other post-school education	29	3.7%

**Awareness of the NHI:** an overwhelming 80.3% of the respondents said they had heard of the NHI while 19.7% said they had not. Almost half (49.4%) who had heard about the NHI, said they heard or obtained information about the NHI from electronic media such as radio or television. More than a third, 38.3% said they heard or obtained information from a community organisation, 7.1% from print media, 4.5% from other sources and 0.6% from billboards.

**Knowledge of how the concept of NHI was developed in South Africa:** overall, a quarter (24.7%) of respondents had knowledge of how the concept of NHI was developed in South Africa while more than two-thirds (71.8%) did not have knowledge of how the concept of NHI was developed in South Africa (Table 2). A quarter of respondents (25.2%) had knowledge of the meaning of “health insurance” in the NHI while 17.9% did not. The latter wrongly said it was the insurance that provided financial help to a family when someone had passed away or covered the costs of the funeral. More than half (56.9%) said they did not know the meaning of “health insurance” in the NHI. Almost half (48.1%) knew that the NHI Fund would pay for medical expenses if a person got sick, while exactly the same proportion (48.1%) did not know; 45.7% knew that with health insurance, basic health requirement is ensured and that if one becomes ill, medical treatment will be paid by the NHI Fund, while 52% did not know and 2.3% were incorrect (Table 3).

**Table 2:** Knowledge of how the concept of NHI was developed in South Africa

	True	False	Don't know
The development of the NHI has been under discussion for many years	24.4%	2%	73.5%
A scheme for a National Health Service for South Africa was first discussed in the 1940s	20.1%	4.7%	75.2%
The current National Health reform has its origins in the ANC health plan of 1994, which included the introduction of a mandatory insurance system	29.5%	3.7%	66.8%
**Average**	**24.7%**	**3.5%**	**71.8%**

**Table 3:** Knowledge of the meaning of “Health Insurance” in the NHI concept

	True	False	Don't know
It is the insurance that provide financial help to your family when someone passes away or cover the costs of the funeral	17.9%	25.2%	56.9%
The NHI Fund will pay for your medical expenses if you get sick	48.1%	3.8%	48.1%
With health insurance, your basic health requirement is ensured; this means that if you become ill, your medical treatment will be paid by the NHI Fund.	45.7%	2.3%	52.0%

**Knowledge of what the NHI is all about:** overall, 52.4% of the respondents had knowledge of the NHI modalities while 44.6% did not know, 3.1% were wrong. Of those who had knowledge of the NHI system, 49.4% knew that NHI was a system that would use funds collected from general taxation to ensure that all citizens were provided with free healthcare, 49.6% knew that under NHI, healthcare will be provided to all, whether employed or unemployed. More than half (51.3%) knew that NHI was designed to enable South African citizens to receive good quality healthcare at any time they required it and 48.5% knew that NHI will allow people to have equal and fair access to skilled health professionals and finance for healthcare. More than half (53.3%) of the respondents knew that both the rich and the poor would receive proper healthcare of the same quality, 49.6% knew that the NHI Fund belonged to all South African citizens, therefore, hospitals and health care professionals could be paid for the service they provide while 54.9% knew that the NHI is government's plan to take care of everyone's health. Majority (62.3%) of respondents knew that the NHI would take 14 years to be fully implemented (Table 4).

**Table 4:** Knowledge of what the NHI is about

	True	False	Don't know
NHI is a system that will use funds collected from general taxation to ensure that all citizens are provided with free healthcare	49.4%	2.2%	48.5%
Under NHI, healthcare will be provided to all, whether you are employed or unemployed	49.6%	4%	46.4%
NHI is designed to enable South African citizens to receive good quality healthcare at any time they require it	51.3%	2.2%	46.5%
NHI will allow people to have equal and fair access to skilled health professionals and equal and fair access to finance for healthcare	48.5%	3.2%	48.3%
Both the rich and the poor will receive proper healthcare of the same quality	53.3%	4.7%	41.9%
NHI Fund will belong to all South African citizens so hospitals and health care professionals can be paid for the service they provide	49.6%	2.4%	48%
The NHI is government's plan to take care of everyone's health in the future.	54.9%	1.4%	43.7%
It will take 14 years to fully implement the NHI	62.3%	4.3%	33.4%

**Knowledge of why we need the NHI:** approximately 59.8% respondents knew that access to healthcare was a human right and that through the NHI it was possible for all South Africans to have access to healthcare, while 38.4% did not know their constitutional right and 56.3% knew that the NHI would provide a fair and equal healthcare system to all South Africans while 41.2% did not know this. Just over half of the respondents (51.8%) knew that because all healthcare needs will be paid for through the NHI, it means the quality of services will be the same throughout the whole country while 45% were not aware. More than half (54%) knew that through the NHI, South Africans will be healthier as more primary health work will be done to prevent illness and people will receive treatment at early stages of illness while 43.4% did not know, 54.5% knew that both the rich and the poor will have more choices of health services under NHI while 41.7% did not know. Only 37.5% knew that the NHI will try to ensure that the sickest people would receive the largest share of healthcare while 44.1% did not know and 18.4% were incorrect (Table 5).

**Table 5:** Knowledge of why South Africa needs the NHI

	True	False	Don't know
Our constitution states that access to healthcare is a human right. Through the NHI, it is possible for all South Africans to have access to healthcare	59.8%	1.8%	38.4%
The NHI will provide us with a health care system that is fair and equal for all South African citizens.	56.3%	2.6%	41.2%
Because all healthcare needs will be paid for through the NHI, it means the quality of services will be almost the same throughout the whole country.	51.8%	3.2%	45%
Through the NHI, South Africans will be healthier as more will be done to prevent illness and people will receive treatment at early stages of illness	54%	2.7%	43.4%
Both the rich and the poor will have more choice of health services under NHI	54.5%	3.8%	41.7%
The NHI will try to ensure that the richest people not the sickest should receive the largest share of healthcare	37.5%	18.4%	44.1%

**Knowledge of how the NHI will work:** overall, 49.8% of respondents did not have knowledge of how the NHI will work. Less than half, 47.1% knew that all NHI patients will enter the healthcare system at primary healthcare level (clinic level or General Practitioner (GP) level). The primary healthcare professional would then refer the patient to a specialist or hospital if needed. This is less than 50.1% who did not know. Nearly a quarter (46.9%) of respondents knew that the same standard of care was expected from private and public healthcare providers as NHI will be expected to deal with high standard service providers, whereas 48.7% did not know. Less than half (46.8%) of respondents knew that the government will upgrade hospitals and other healthcare facilities to ensure that the standards of these facilities were improved or good enough to satisfy the standard expected by the NHI compared to 49.6% who did not know. Almost a quarter (46.9%) of respondents knew that money was being spent on training of health care professionals since with good infrastructure and well skilled professionals; the NHI would be successful while 50.1% did not know. More than half of the respondents (52.7%) knew that the NHI will improve preventive healthcare and care in the early stages of illness. This is more than 44.6% who did not know. Only 43.5% knew that the NHI will introduce family health teams in neighborhoods which will provide preventative health services and home-based care, such as nurses, community doctors and home-based carer providers while 52.4% did not know. Only 44.1% knew that the government had started to implement the NHI in pilot sites to determine how it will work, while 53.3% did not know.

### Who should pay for the NHI?

Less than a third (29.8%) incorrectly thought it would be possible to opt out of the NHI, while 49.6% thought this was not possible. Interestingly, 54.5% understood that a large amount of the funding would come from general taxes, therefore, all tax payers would contribute to the NHI while 43.2% did not know this. Just over half of respondents (51.7%) knew that all permanent employees would be expected to contribute to the NHI fund as compared to 44.1% who did not know. Only 42.7% knew that every person who earns over a certain specified amount would be required by law to make a special monthly payment to the NHI Fund (called the “NHI contribution”) as compared to 53.3% who did not know. Additionally, 41.9% of respondents understood that employers would be expected to cooperate with NHI to ensure legible tax payers make their NHI contributions, similar to pension funds, while 53.6% did not. Only 40.3% understood that employers will also match their employees’ contributions by paying a monthly amount to the NHI, for every worker, while 54.6% did not.

## Discussion

The level of awareness regarding the NHI in this study was very high. Approximately 84.6% of the respondents were in the age range of 20-59 years. This was less than the 93.3% of respondents who were between 25 and 65 years of age who participated in a similar study in Nigeria [11]. Only 21% of respondents in this study were married, while 73.4% were single. This is comparable to the 76.7% of respondents who were married in a study conducted by Lawan, et al. in Nigeria [11]. In this study, 80.3% of the respondents had heard of the NHI. Evans and Shisana [12] found that awareness of the NHI in South Africa was very high with 90.8% respondents expressing that the NHI should be a national priority and over 80% saying they would prefer it to the current healthcare system. Awareness about the NHI in South Africa exceeded that in Uganda, where only 40.7% had heard about the proposed Social Health Insurance scheme and more than a half of the respondents (57.3%) had never heard about it [13]. This post-test level of awareness about NHI might be attributed to the work of the Monash-Oxfam NHI project and their collaborating Partners who conducted community consultation processes to raise awareness about the NHI in these areas. In this study, 49.4% of the respondents reported that they heard or became aware of the NHI from electronic media such as radio or television, 38.3% said they heard or got information from a community organisation, 7.1% from print media, 4.5% from other sources and only 0.6% from billboards. This is slightly higher than a Ugandan study where 38% reported that they had read about it in local newspapers, 26.9% from fellow staff, 20.4% from radios, 7.4% from television, only 4.6% from both television and radios and 2.8% from workshops [13]. Knowledge about what the NHI was all about was generally poor. In this study 44.6% of the respondents did not know what the NHI was all about. This is somewhat lower than 52% of the respondents in Nigeria who had poor knowledge of NHIS [11]. In this study, less than half (49.8%) respondents did not have knowledge of how the NHI will work. This is comparable to findings by Lawan et al [11] where less than half of the respondents in Nigeria knew the objectives of the NHIS, the ways of enrolling in the scheme, or the amounts to be paid as premium by the employer and employee. Again, 71.8% of respondents in this study did not know how the concept of NHI was developed in South Africa.

The knowledge of what the NHI would pay for in this study was also poor. This was surprising as 75.6% of respondents were unemployed and should have wanted to know how the NHI would benefit them. Less than half (48.1%) knew that the NHI fund would pay for medical expenses if a person got sick, and 45.7% knew that with health insurance, basic health requirement is ensured and that if one becomes ill medical treatment would be paid by the NHI Fund. It was encouraging that 54.5% of the respondents understood that a large amount of the funding would come from general taxes, so all tax payers would contribute to the NHI and 51.7% knew that all permanent employees would be expected to contribute to the NHI fund. These findings show that public awareness campaigns about the NHI focused more on what the NHI would pay on behalf of users. The findings in this study on understanding what the NHI would pay for were far lower than in a study conducted in Kumasi, Ghana, where 97.9% of the clients interviewed had heard of capitation payment. Similar to this study, high level of awareness did not translate into higher level of knowledge [9]. The disappointing findings were that 29.8% incorrectly thought it would be possible to opt out of the NHI, if they had not voted for the present government while 50.9% of respondents did not understand how the NHI Fund will pay for health care received. However, the findings in our study were better than outcome of the Nigerian study where less than one-third of the respondents knew which age-group (among children) was eligible for registration under the scheme, what the special social insurance provisions were, or what services were offered under the NHIS [11]. Awareness and support for NHI in South Africa has grown from 56.9% in 2005 [14] to 73.2% in 2008 [15] to 80% in 2012 [12] and was found to be 80.3% in this study. This overwhelming support is not surprising, given the challenges presented by the public health care system and the high cost of private healthcare. This was confirmed by McIntyre et al [15] who found that the majority of South Africans were dissatisfied with the current healthcare system and thought private health care was expensive. Awareness and support for publicly funded healthcare systems is high in other middle-income countries ranging from 70% in Taiwan [16], over 85% Canada [17] and over 89% in Thailand [18]. Similarly, most Australians support Medicare over holding down taxes [19]. These examples are encouraging for South Africa, as they suggest that awareness and public support for NHI will continue well beyond the NHI pilot phase.

## Conclusion

The study showed that the levels of awareness of the NHI were high in the first two years of its introduction in South Africa. However, knowledge of what the NHI was all about was generally poor. Public awareness campaigns about the NHI were generally good while the education campaigns to increase knowledge and understanding of the scheme were narrow and did not penetrate many communities where information about the NHI was sought. Public awareness campaigns about the NHI focused more on what the NHI would pay on behalf of users. The understanding of what the NHI would pay for in this study was reasonable overall. The overwhelming awareness and support for the NHI must be used to mobilise communities and to increase knowledge and change any negative attitudes about the scheme. Using an information brochure to increase knowledge about a new initiative such as the NHI is not sufficient to achieve that objective.
